# *Enterococcus faecalis* bloodstream infection: does infectious disease specialist consultation make a difference?

**DOI:** 10.1007/s15010-021-01717-3

**Published:** 2021-10-29

**Authors:** Chiara Cattaneo, Siegbert Rieg, Guido Schwarzer, Matthias C. Müller, Benjamin Blümel, Winfried V. Kern

**Affiliations:** 1 Division of Infectious Diseases, Department of Medicine II, Medical Center, Faculty of Medicine, University of Freiburg, Freiburg, Germany; 2 Institute of Medical Biometry and Statistics, Faculty of Medicine and Medical Center, University of Freiburg, Freiburg, Germany; 3 Institute of Medical Microbiology and Hygiene, Faculty of Medicine, Medical Center-University of Freiburg, Freiburg, Germany

**Keywords:** *E. faecalis*, Bloodstream infection, ID consultation

## Abstract

**Purpose:**

To evaluate the relationship between mortality or relapse of bloodstream infection (BSI) due to *Enterococcus faecalis* and infectious diseases specialist consultation (IDC) and other factors potentially associated with outcomes.

**Methods:**

In a tertiary-care center, consecutive adult patients with *E. faecalis* BSI between January 1, 2016 and January 31, 2019, were prospectively followed. The management *of E. faecalis* BSI was evaluated in terms of adherence to evidence-based quality-of-care indicators (QCIs). IDC and other factors potentially associated with 90-day-mortality or relapse of *E. faecalis* BSI were analyzed by multivariate logistic regression.

**Results:**

A total of 151 patients with a median age of 68 years were studied. IDC was performed in 38% of patients with *E. faecalis* BSI. 30 cases of endocarditis (20%) were diagnosed. All-cause in-hospital mortality was 23%, 90-day mortality was 37%, and 90-day relapsing *E. faecalis* BSI was 8%. IDC was significantly associated with better adherence to 5 QCIs. Factors significantly associated with 90-day mortality or relapsing EfB in multivariate analysis were severe sepsis or septic shock at onset (HR 4.32, CI 2.36e7.88) and deep-seated focus of infection (superficial focus HR 0.33, CI 0.14e0.76).

**Conclusion:**

*Enterococcus faecalis* bacteremia is associated with a high mortality. IDC contributed to improved diagnostic and therapeutic management.

## Introduction

*Enterococcus* spp. are important causes of both community- and hospital-acquired bloodstream infections and account for 10% of endocarditis cases worldwide [1]. Incidence of *E. faecalis* BSI (EfB) has been estimated to be ~ 4.5 per 100,000 annually and case fatality between 10 and 20% [2, 3].

EfB has been described as a clinical entity with different risk factors, clinical features, and microbiological characteristics than other enterococcal BSIs [2]. Specifically, EfB occurs in less seriously ill patients, has lower rates of antibiotic resistance, and is more often associated with endocarditis. *E. faecalis* causes both native valve and prosthetic valve endocarditis, while *E. faecium* endocarditis remains a rare entity [1].

The optimal management of *E. faecalis* invasive infections is still not clearly defined and data concerning the risk of developing endocarditis are divergent. In a recent study, Dahl et al. found that infective endocarditis (IE) was diagnosed 1 out of 4 patients with EfB who systematically received echocardiography [4].

Infectious diseases specialist consultation (IDC) as a strategy to improve the quality of care has been investigated in other difficult-to-treat infections such as *Staphylococcus aureus* bloodstream infection and candidemia. In these studies, IDC led to better adherence to diagnostic and management standards, and was associated with lower mortality [5–12].

Recent investigations described the role of IDC in management of enterococcal BSI, suggesting that IDC consultations are associated with a greater likelihood of elimination of bacteremia [13] and improved outcome [14–16]. In the present paper, we evaluated the epidemiology, clinical characteristics, and outcomes of patients with EfB admitted to our hospital between January 1, 2016, and January 31, 2019, with a focus on the impact of ID specialist consultation on diagnostic work-up and outcome.

## Patients and methods

### Setting, case identification, and study design

The study was conducted from January 1, 2016, to January 31, 2019, at the Medical Center of the University of Freiburg, Germany, a 1600-bed tertiary-care center with 73,000 admissions and 824,000 outpatient contacts per year. Consecutive adult patients with clinical evidence of infection and blood cultures growing *E. faecalis* were prospectively included and followed for at least 90 days. Identification and susceptibility testing of all blood culture isolates was performed according to standard protocols.

### Data collection

The following data were recorded for each patient: age, sex, underlying disease, comorbidity according to the Charlson Comorbidity Index, date of hospital admission, stay or admission to an intensive care unit (ICU), and clinical parameters at EfB onset. Initial portal of entry of EfB, main focus, diagnostic investigations, antimicrobial therapy, and outcomes were documented. The data were retrieved directly or electronically from clinical charts and reports including diagnostic (e.g., laboratory, microbiological, or radiographic) studies. All patients were followed during their hospital stay and for at least 90 days after onset. Mortality, hospital readmissions, and relapsing infection were assessed by reviewing medical records of our institution and/or seeing the patient in the infectious disease (ID) outpatient department.

The Institutional Review Board of the University Medical Center Freiburg considered the collection of routine data as evaluation of service and waived the need for written informed consent.

### Definitions

The day of sampling the first blood culture positive for *E. faecalis* was considered the date of EfB onset. Community-acquired EfB was considered as EfB diagnosed before, at, or within 48 h after hospital admission. Healthcare-associated EfB was defined as EfB within 48 h of admission in an outpatient with (1) close healthcare contact or (2) hospitalization for 2 or more days within 90 days or (3) continuous residency in a nursing home before EfB. All other EfB cases were defined as hospital acquired. ICU admission was defined between 24 h before and 48 h after onset. The focus of infection was defined as a focal infection with typical signs or symptoms of infection, isolation of *E. faecalis* at the site of infection, and/or imaging results compatible with focal infection. Catheters were considered as the source of EfB if both central line and peripheral blood cultures were growing *E. faecalis*, and catheter tip cultures grew *E. faecalis* as well. Endocarditis was diagnosed according to the modified Duke criteria [17]. Severe neutropenia was defined as an absolute neutrophil count of less than 500/mL. We considered severe immunosuppression in the presence of primary immunodeficiency disorders, uncontrolled disease in HIV-positive patients, high-dose steroid therapy, and immunosuppressive combination therapy with two or more drugs with different mode of action, hematopoietic stem cell transplantation within the past 6 months, and solid organ transplant. Infections of a removable catheter and uncomplicated urinary infections were considered as superficial foci. Biliary tract and other intraabdominal infections, spondylodiscitis, osteomyelitis, and endocarditis were considered deep-seated foci. Embolization was defined by signs on clinical examination or by findings using imaging techniques. EfB were considered complicated if any of the following criteria were present: severe sepsis/septic shock within 24 h before or after onset, persistent positive blood cultures, presence of deep-seated focus (for example, undrained abscess, empyema, and osteomyelitis) or endocarditis, need of surgical or non-invasive intervention, presence of a non-removable foreign body or an intravascular device (prosthetic joint, prosthetic heart valve, implantable electronic cardiac devices, long term intravascular catheter, and vascular prosthesis), and local spread of infection. Persistent positive blood cultures were defined as the continued presence of *E. faecalis* in blood cultures at day 3 or later after beginning of effective therapy. Relapse of bacteremia was defined as the isolation of *E. faecalis* (with the same resistance profile) from blood cultures within 90 days after EfB onset and following end of treatment for the initial episode.

### Infectious disease (ID) specialist consultation and quality-of-care indicators

An ID service has been established in our hospital since 2002. IDCs for EfB are performed on request of the primary physician in charge. IDC comprised chart review and physical examination of the patient with written recommendations for diagnostic work-up, therapy, and follow-up examinations based on established guidelines, literature review, and individual case discussions with the physicians in charge. During the 3-year study period, senior consultants of the ID service did not change. To evaluate the impact of IDC on the management of EfB, we identified five evidence-based quality-of-care indicators (QCIs) based on clinical experience and extrapolated from the literature [14, 15, 18], which were defined according to Table 1.

**Table 1:** Definition of quality-of-care indicators

Quality of care indicator	Definition
Follow-up blood cultures	Performance of control blood cultures 48–96 h after onset of bacteraemia, regardless of clinical evolution
Early source control	Removal of non-permanent vascular catheter whenever the catheter was suspected or confirmed as the source of EfB, or eradication/drainage of a deep-seated focus (e.g., drainage of abscess, stent placement in case of ureter or biliary obstruction) within 48–72 h
Echocardiography in patients at risk of endocarditis	Performance of echocardiography especially in patients with complicated bacteraemia or other predisposing conditions for endocarditis such as community-acquired EfB or unknown origin of bacteraemia
Appropriate definitive therapy	Definitive therapy with intravenous ampicillin (at least 2 g every 6 h or adjusted to renal function ^a^), or Definitive combination therapy in patients with endocarditis, endovascular prosthesis infection or osteomyelitis, or Definitive therapy with the narrowest antimicrobial spectrum in case of polymicrobial bacteraemia^b^, started within 72–96 h after EfB onset
Treatment duration according to the complexity of infection	Duration of antimicrobial therapy for 3–7 days for uncomplicated bacteraemia, 7–14 days for uncomplicated bacteraemia with presence of non-removable intravascular device, ≥ 42 days in case of suspected or confirmed endocarditis

^a^Dosing was adjusted to renal function according to the Stanford Health Care Antimicrobial Dosing Reference Guide [19].

^b^Adequate antimicrobial monotherapy was defined as administration of a cell wall-active agent [a b-lactam (agent of first choice: ampicillin)]. Combination therapy was defined as adequate monotherapy plus additional administration of gentamicin (standard dose 3 mg/kg/24 h i.v. or i.m. or adjusted to renal function) or ceftriaxone (2 mg/12 h i.v. or i.m. or adjusted to renal function). For patients with polymicrobial bacteremia, adequate antimicrobial therapy was defined as administration of daptomycin, linezolid, ampicillin/sulbactam, or piperacillin/tazobactam. In polymicrobial bacteremia with the presence of *S. aureus*, additional therapy with flucloxacillin or cefazolin was considered adequate

### Study endpoints

The primary endpoint of the study was death or relapsing EfB within 90 days. Secondary endpoint was all-cause in-hospital mortality and the adherence to quality-of-care indicators in EfB patients.

### Statistical methods

Continuous variables were summarized with medians and interquartile ranges (IQR) and categorical variables with frequencies and percentages. Proportions were compared using the Pearson *χ*^2^, medians using the Wilcoxon rank‐sum test.

We investigated the effect of various patient characteristics and IDC on mortality or relapse of EfB 90 days after onset. The hazard risk for death or relapsing infection [along with the 95% confidence interval (CI)] was estimated with a Cox regression with inclusion of variables known to be relevant for the outcome. A sensitivity analysis was conducted using a propensity score-adjusted Cox regression. The propensity score for ID consultation was based on age, sex, Charlson score, ICU admission at onset, mode of acquisition, endocarditis or deep-seated focus, and presence of intravascular device or foreign body. In addition, severe sepsis or septic shock at onset was included in the Cox regression. ID consultation was expressed as time-dependent covariable for avoiding immortal time bias. A *p* value of < 0.05 was considered statistically significant in all analyses.

Statistical analyses were performed with IBM SPSS Statistics 25.0 and STATA version 12.1 (StataCorp; College Station, TX, United States of America).

## Results

### Clinical features

**Fig. 1:**
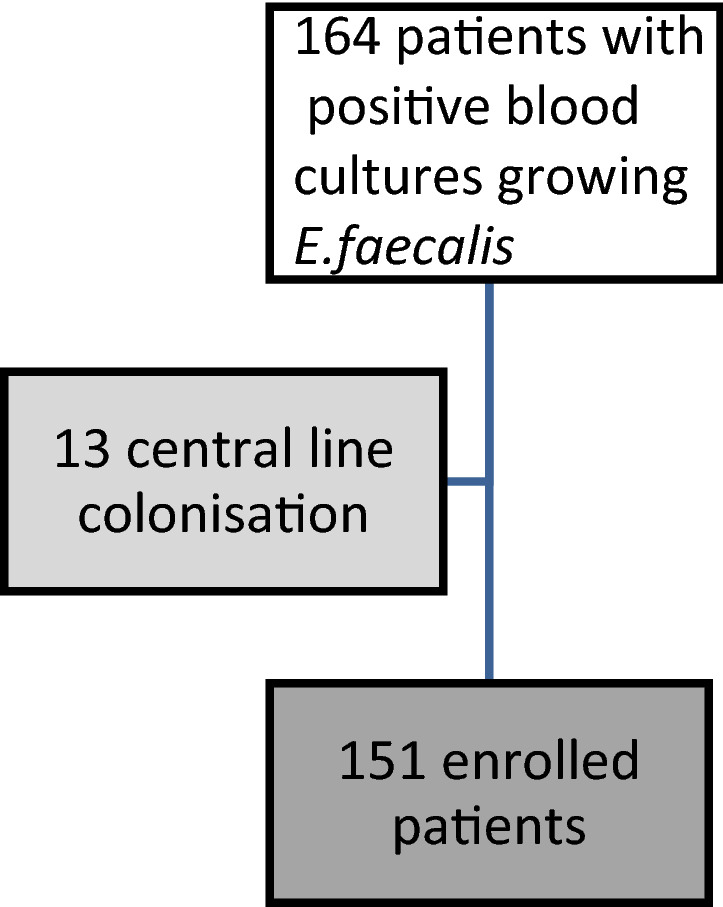
Study flowchart

As shown in the study flowchart (Fig. 1), 164 consecutive adult patients with clinical evidence of infection and blood cultures growing *E. faecalis* were identified during the 3-year period. Patients with central line colonization (*n* = 13) without a positive peripheral blood culture and no sign or symptoms of infection were not included. A total of 151 patients with a median age of 68 years were eligible for inclusion in the study. Patient characteristics are shown in Table 2. 60 (40%) patients suffered from polymicrobial bloodstream infections. 64 (42%) required ICU admission. Malignancies including hematological and solid tumors (65, 43%), chronic renal failure (45, 30%), and diabetes mellitus (39, 26%) were the most common underlying conditions. 78 (52%) of infections were hospital-acquired and 34 (23%) healthcare-related. All 151 *E. faecalis* isolates were susceptible to ampicillin and vancomycin. High-level gentamicin resistance was detected in 45 (30%) isolates. In 36 (24%) patients, the portal of entry was unknown. Among the hospital-acquired infections, intravascular devices, and surgical interventions (23%, respectively) were the most common source of EfB. Healthcare-associated and community-acquired infections showed high rates of biliary tract (27% and 20%, respectively) and urinary tract infections (27% and 15%, respectively) (data not shown). 30 cases of endocarditis (20%) were diagnosed.

**Table 2:** Epidemiological, clinical characteristic, and outcomes of 151 patients with *E. faecalis* bacteremia

Variable	All patients *n* = 151	ID consultation *n* = 58	No ID consultation *n* = 93	*p* value
Median age (IQR)	68 (59–78)	72,5 (60–80)	68 (57–77)	0.12
Female sex	41 (27%)	11 (19%)	30 (32%)	0.07
Charlson index ≥ 5	37 (24%)	18 (44%)	19 (20%)	0.14
ICU admission	64 (42%)	23 (40%)	41 (44%)	0.59
Polymicrobial BSI	60 (40%)	19 (32%)	41 (44%)	0.17
Underlying conditions				
^Diabetes mellitus	39 (26%)	18 (31%)	21 (23%)	0.22
^Chronic renal disease	45 (30%)	23 (40%)	22 (24%)	0.04
^Chronic liver disease	15 (10%)	5 (9%)	10 (11%)	0.25
^Malignancy	65 (43%)	15 (26%)	50 (54%)	0.002
^Pacemaker	20 (13%)	13 (22%)	7 (8%)	0.009
^Prosthetic heart valve	25 (17%)	18 (31%)	7 (8%)	< 0.001
^Intravenous drug abuse	4 (3%)	2 (3%)	2 (2%)	0.63
^Severe neutropenia	2 (1%)	0	2 (2%)	1.30
^Severe immunosuppression	20 (13%)	6 (10%)	14 (15%)	0.40
Acquisition				
^Hospital-acquired	78 (52%)	26 (45%)	52 (56%)	0.18
^Community-acquired	39 (26%)	23 (39%)	16 (17%)	0.001
^Healthcare-associated	34 (23%)	9 (16%)	25 (27%)	0.06
Complicated bacteraemia^a^	106 (70%)	50 (86%)	56 (60%)	0.001
^Hemodynamic instability/shock	28 (19%)	10 (17%)	18 (19%)	0.75
^Persistent positive blood culture	15 (10%)	11 (19%)	4 (4%)	0.004
^Endocarditis or deep-seated focus	82 (54%)	39 (67%)	43 (46%)	0.045
^Intravascular device or foreign body	39 (26%)	26 (45%)	13 (14%)	< 0.001
Portal of entry				
^Unknown	36 (24%)	25 (43%)	11 (12%)	< 0.001
^Intravascular device	25 (17%)	11 (19%)	14 (15%)	0.53
^Abdominal surgery	24 (16%)	3 (5%)	21 (23%)	0.004
^Biliary tract or stent	23 (15%)	5 (9%)	18 (19%)	0.07
^Urinary tract	21 (14%)	3 (5%)	18 (19%)	0.07
^Gastrointestinal tract	15 (10%)	9 (16%)	6 (6%)	0.14
^others	7 (4%)	2 (3%)	5 (5%)	0.79
Main focus				
^Unknown	23 (15%)	9 (15%)	14 (14%)	0.94
^Intravascular device	20 (13%)	7 (12%)	13 (14%)	0.74
^Endocarditis	30 (20%)	30 (52%)	0	< 0.001
^Left (native)	14 (47%)	14 (47%)		
^Left (prosthetic)	12 (40%)	12 (40%)		
^Right (native)	1 (3%)	1 (3%)		
^Pacemaker	3 (10%)	3 (10%)		
^Osteomyelitis/	3 (2%)	1 (2%)	2 (2%)	0.85
^Spondylodiscitis				
^Biliary tract/stent	25 (17%)	5 (9%)	20 (22%)	0.38
^Other intraabdominal focus	24 (16%)	3 (5%)	21 (23%)	0.004
^Urinary tract	21 (14%)	2 (3%)	19 (20%)	0.003
^Others	5 (3%)	1 (2%)	4 (4%)	0.39
Outcome				
All-cause in-hospital mortality	35 (23%)	12 (21%)	23 (25%)	0.57
90-day mortality	43 (37%)	16 (37%)	27 (38%)	0.95
Relapse of *E. faecalis* BSI	9 (8%)	0	9 (13%)	0.05

^a^More than one complication is possible in each patient

Overall, ID physicians were involved in 58 (38%) of EfB cases. IDC were performed during the first week after EfB onset (median 3 days). Patients in the IDC group had more frequently chronic renal failure, higher presence of pacemaker, or prosthetic heart valve, and suffered more frequently from complicated bacteremia (86% vs 60%). Malignancy was more frequently found in the non-IDC group (54 vs 26%) (Table 2).

### Outcomes

All-cause in-hospital mortality was 23% (35 of 151 patients). Of the patients who survived, 48% were discharged home, 25% were transferred to other medical facilities, and 3% were discharged to a nursing home. 36 patients were lost to follow-up before day 90 after onset. Death within 90 days after EfB onset was reported in 43 of the 115 evaluable patients (37%). No significant difference of in-hospital and 90-day-mortality was observed between the IDC and no-IDC group. Relapsing within 90 days was observed in 9 patients (8%), all of which in the non-IDC group.

### Adherence to quality-of-care indicators

As shown in Table 3, follow-up blood culture (91% vs. 52%, *p* < 0.001) and echocardiography (84% vs. 25%, *p* < 0.001) were more frequently performed in the IDC group. The source of bacteremia was better controlled in patients followed by an ID specialist (89% vs 65%, *p* = 0.001). In the IDC group, early appropriate definitive therapy was more frequently prescribed (78% vs 30%, *p* < 0.001) and the duration of treatment was more frequently appropriate according to the complexity of infection (76% vs 43%, *p* = 0.001).

**Table 3:** Adherence to quality-of-care indicators in 151 patients with E. faecalis bacteremia, with and without ID consultation

Quality-of-care indicators	Tot	ID consultation *n* = 58	No ID consultation *n* = 93	RR for adherence to QCI (95% CI)	*p* value
Follow-up blood cultures	105	53 (91%)	52 (56%)	1.63 (1.34–1.99)	**< 0.001**
Early source/focus control	100	50 (89%)	50 (65%)	1.37 (1.14–1.66)	**0.001**
Echocardiography	72	49 (84%)	23 (25%)	3.41 (2.36–4.95)	**< 0.001**
Use of intravenous ampicillin as definitive therapy	72	45 (78%)	27 (30%)	2.61 (1.85–3.69)	**< 0.001**
Adequate treatment duration	83	44 (76%)	39 (43%)	1.77 (1.34–2.35)	**0.001**

A* p* value of < 0.05 was considered statistically significant

### Factors associated with death or recurrence within 90 days

To evaluate the impact of factors potentially associated with death or relapse of EfB, a univariate and a multivariate analyses were performed in a Cox regression model; results are shown in Table 4. Severe sepsis or septic shock within 24 h before or after onset (HR 4.05, CI 2.30–7.15; HR 4.32, CI 2.36–7.88) and deep-seated focus of infection (superficial focus HR 0.38, CI 0.17–0.86; HR 0.33, CI 0.14–0.76) were significantly associated with mortality and recurrence of bacteremia within 90 days, both in univariate and multivariate analyses. The mortality was higher in patients with hospital-acquired and healthcare-associated EfB, but the difference was not significant. Also, IDC was associated with survival or relapse of EfB with an adjusted HR of 0.87 (0.45–1.66) that was not statistically significant. The propensity score-adjusted Cox regression supported the results of the multivariate analysis and did not show an impact of IDC on mortality (Table 5).

**Table 4:** Univariate and multivariate analyses of characteristics and factors potentially associated with death or relapse within 90 days in 151 cases of *E. faecalis* bacteremia

Variable	Subgroup	Mortality or recurrence of bacteraemia after 90 days					
		Univariate analysis			Multivariate analysis		
		HR	95% CI	*p* value	HR	95% CI	*p* value
Sex	Male	1			1		0.87
	Female	0.95	0.52–1.75	0.87	0.95	0.50–1.80	
Age (years)		1.004	0.99–1.03	0.63	1.01	0.99–1.04	0.30
Charlson Score ≥ 5		1.27	0.70–2.31	0.44	1.00	0.5–1.98	0.99
Severe sepsis or septic shock at onset		4.05	2.30–7.15	< 0.001	4.32	2.36–7.88	< 0.001
Main infection focus				0.031			0.027
	Deep-seated focus	1			1		
	Unknown focus	1.004	0.47–2.16	0.99	1.18	0.49–2.82	0.72
	Superficial focus	0.38	0.17–0.86	0.02	0.33	0.14–0.76	0.009
Mode of acquisition				0.44			0.19
	Community-acquired	1			1		
	Hospital-acquired	1.31	0.66–2.61	0.43	1.37	0.62–3.01	0.44
	Healthcare-associated	1.64	0.76–3.51	0.20	2.14	0.92–5.00	0.08
ID specialist consultation		0.92	0.49–1.73	0.80	0.87	0.45–1.66	0.66

IDC is expressed as time-dependent covariable

**Table 5:** Propensity score-adjusted Cox regression. IDC is expressed as time-dependent covariable

Variable	Multivariate analysis		
	HR	95% CI	*p* value
Propensity score^a^	0.67	0.20–2.24	0.52
Severe sepsis or septic shock at onset	4.00	2.25–7.07	< 0.001
ID specialist consultation	0.95	0.50–1.79	0.87

^a^The propensity score for ID consultation was based on age, sex, Charlson score, ICU admission at onset, mode of acquisition, endocarditis or deep-seated focus, and presence of intravascular device or foreign body

## Discussion

The key findings of this study were that among EfB patients severe sepsis/septic shock and deep-seated infection are strongly associated with mortality or relapse, whereas IDC as performed in our center resulted in improved diagnostic management and antimicrobial therapy, but its impact on 90-day mortality or relapse was uncertain.

Clinical epidemiological findings in this study were comparable to those reported in the other studies from different parts of the world [2, 3, 20, 21]. In our study, we found a higher frequency of infectious endocarditis (IE) (20%) than in some previous studies [3, 15, 22] but comparable to recent findings of a Danish study reporting endocarditis in 26% of the EfB patients [4]. Fernández-Hidalgo et al. reported an increase of Ef-IE of 10% from 2000 to 2018, with Ef-IE accounting for 23% of all endocarditis cases [22]. These results show that patients with EfB are at high risk of IE and underline the importance of performing echocardiography, especially in patients with predisposing conditions for IE, unknown focus, and persistent positive blood cultures. Our case collection was based in a tertiary-care center with an endocarditis team and a cardiovascular surgery department; this may have introduced a referral bias toward a selection of more severe cases. This may also explain the slightly higher mortality compared to several previous studies [2, 3].

An ID service has been established in our hospital since 2002; in 2018, ~ 2000 consultations were performed by our consultation service. During the period of the study, ID physicians were involved in 38% EfB cases on request of the primary physician in charge. Many previous studies evaluated the role of an expert consultation service in *S. aureu*s bacteremia, where the high risk of IE and invasive infection is well known [23–27]. A previous work from our group suggested better diagnostic management, more adequate treatment, and lower mortality rate of patients with *S. aureus* bacteremia followed by IDC [5]. In this study, IDC significantly contributed to better therapeutic decision, but did not significantly impact on 90-day mortality or EfB-relapse in the multivariate analysis, even though relapsing EfB was observed only in the non-IDC group. A propensity score for ID consultation was calculated considering demographic factors (age, sex), comorbidities (Charlson score), ICU admission at onset as a proxy of severity of illness, and factors that showed a statistical different distribution among the IDC and non-IDC group. However, the propensity score-adjusted Cox regression supported the results of the multivariate analysis. The most likely explanations are the small study population, further unmeasured confounding, and the high prevalence of complex underlying conditions making survival as an endpoint perhaps insensitive for the range of possible improved outcomes. Previous studies described IE as leading cause of recurrent EfB [15]. In this study, endocarditis was diagnosed in 52% of patients followed by IDC; therefore, the absence of relapse in all IDC-patients is noteworthy. We believe that a better adherence to important practice guidelines and recommendations for management of Ef-IE played a major role.

To our knowledge, a few studies evaluated the impact of IDC on management and outcome in enterococcal bacteremia. A recent study from Italy assessed the impact of ID consultation in 368 patients with monomicrobial enterococcal bacteremia, showing an improved 30-day and 1-year mortality in the IDC group. In accordance to our findings, they reported more frequent adequate therapy, echocardiography, and follow-up blood cultures in patients visited by an ID specialist [15]. Similarly, Lee et al. reported better diagnostic management associated with IDC in 205 patients with enterococcal bacteremia. A lower all-cause 30-day mortality was associated with IDC in this study; however, IDC did not impact on 90-day mortality [16].

A recent investigation from Japan evaluated the impact of IDC on enterococcal bacteremia in children; ID consultations were significantly associated with appropriate empiric and definitive therapy, appropriate treatment duration, and lower risk of 1-year mortality [28]. Zasowski et al. reported that IDC within 24 h was significantly associated with a lower risk for delayed appropriate therapy, and therefore with a lower 30-day mortality in 190 patients with enterococcal BSI [14]. Jindai et al. found that blood culture sterilization occurred more frequently when IDC was obtained [13].

In this study, we specifically focused on management of *E. faecalis* bacteremia, and we evaluated the impact of IDC in terms of quality of diagnostic work-up, quality of therapy, and outcome. As EfB has been less investigated in this respect, we based our choice of quality-of-care indicators on our previous clinical experience and on established recommendations for treatment of EfB. Since both EfB and *S. aureus* BSI are connected with a high risk of endocarditis [1], we extrapolate the QCIs from those established for *S. aureus* infections [18]. Our findings underline that expert advice can lead to a better adherence to guidelines, to a more detailed evaluation, early effective therapy, and appropriate treatment duration.

Of note is that we focused on bloodstream infection due to *E. faecalis* and did not include other enterococci. *E. faecalis* and *E. faecium* are considered two different bacterial species and may be associated with different clinical syndromes. *E. faecium* endocarditis, for example, is rare [2], and *E. faecium* may be associated with different underlying diseases and risk factors. A limitation of this study is its single-center nature. Moreover, some information were missing in clinical charts and reports and some of the relevant data may have been missed during review of the medical records. Patient follow-up after discharge was incomplete, and 36 patients were lost to follow up before day 90 after onset; therefore, we may have missed deaths and recurrences. In addition, the two groups of patients with and without IDC had comparable demographic characteristics, but differed regarding relevant clinical findings and underlying conditions. Polymicrobial bacteremia was included in this study and concomitant bacteria may have contributed to mortality. Finally, we evaluated overall mortality rather than attributable mortality, which, however, is difficult to determine.

## Conclusion

In our study, we observed a relative high mortality of patients with EfB, which may be related to underlying conditions and severe manifestations, as the study was conducted at a tertiary-care referral center. Endocarditis is a frequent condition in patients with EfB and echocardiography should be included in the diagnostic work-up. IDC contributed to an improved diagnostic and therapeutic management of patients with EfB. No relapsing EfB within 90 days was observed in patients followed by an ID specialist.

## Data Availability

Not applicable.
